# Supplementary material for: A multi-scale computational model of the effects of TMS on motor cortex
Source: F1000Res. 2017 May 12;5:1945. Originally published 2016 Aug 10. [Version 3] doi: 10.12688/f1000research.9277.3 (PMC5373428; doi:10.12688/f1000research.9277.3)
Supplement: Supplementary file 5 [file f1000research-5-12345-s0004.tgz › 7ee0b64b-f08d-48be-a27e-7be0cca40c4a.pptx]

## Slide 1
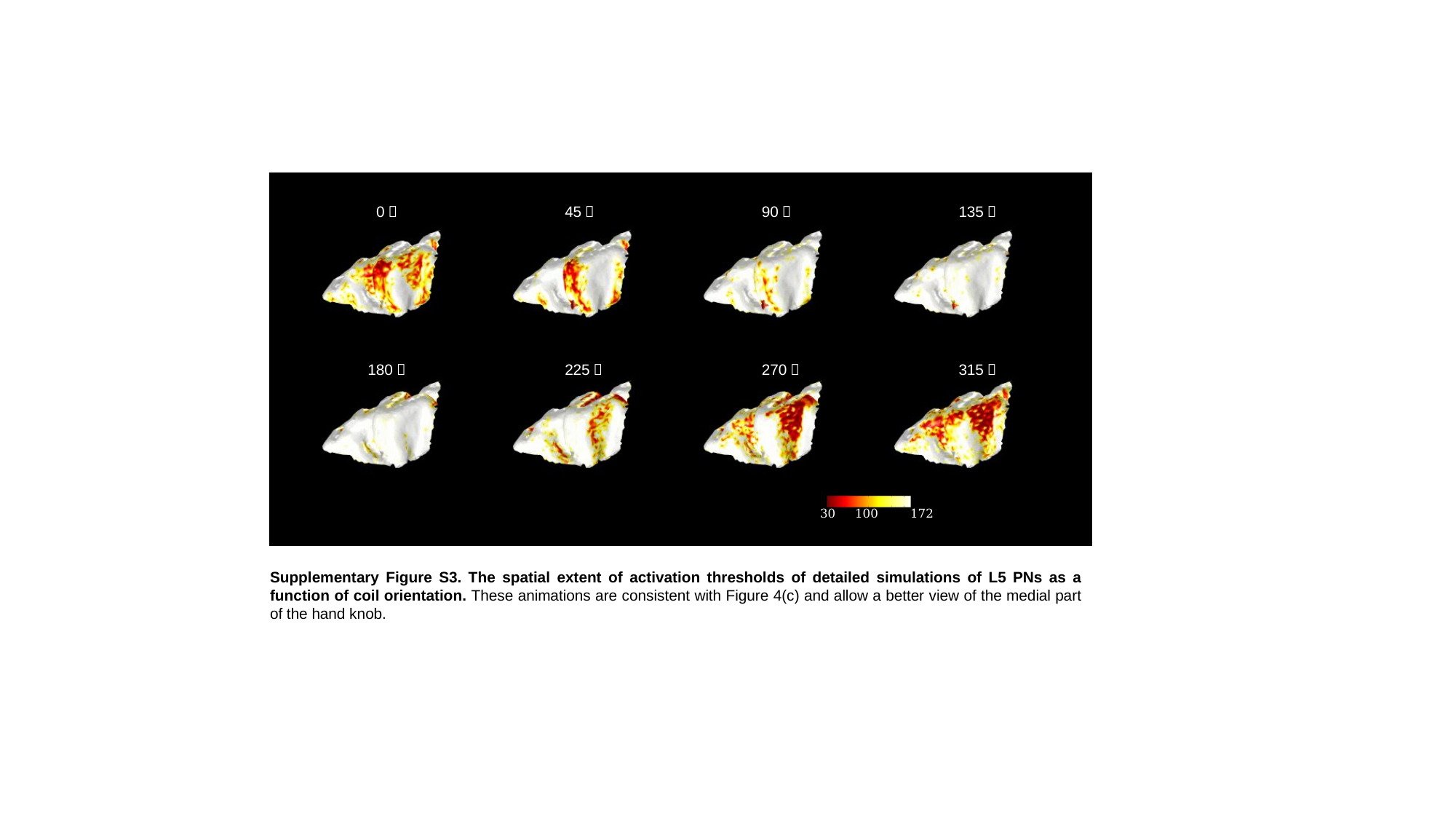

0〫
45〫
90〫
135〫
180〫
225〫
270〫
315〫
30
100
Supplementary Figure S3. The spatial extent of activation thresholds of detailed simulations of L5 PNs as a function of coil orientation. These animations are consistent with Figure 4(c) and allow a better view of the medial part of the hand knob.
