# Supplementary material for: A multi-scale computational model of the effects of TMS on motor cortex
Source: F1000Res. 2017 May 12;5:1945. Originally published 2016 Aug 10. [Version 3] doi: 10.12688/f1000research.9277.3 (PMC5373428; doi:10.12688/f1000research.9277.3)
Supplement: Peer review history

# Peer review: Referee response for version 3

**DOI:** 10.5256/f1000research.12345.r22655

**Published:** 2017-05-31

**Authors:** Socrates Dokos

**Affiliations:** 1 Graduate School of Biomedical Engineering, Faculty of Engineering, University of New South Wales, Sydney, NSW, Australia

In their re-revised article, the authors have adequately addressed the previous points raised by the reviewers. Am happy to recommend acceptance.

I have read this submission. I believe that I have an appropriate level of expertise to confirm that it is of an acceptable scientific standard.

# Peer review: Referee response for version 2

**DOI:** 10.5256/f1000research.10355.r20336

**Published:** 2017-03-24

**Authors:** Axel Thielscher

**Affiliations:** 1 Danish Research Centre for Magnetic Resonance, Copenhagen University Hospital Hvidovre, Hvidovre, Denmark

The revised manuscript was substantially improved. I have two remaining points, which I suggest to clarify in form of a minor revision of the article:

1. At the TBS conference in Göttingen 2016, Peterchev and colleagues presented modelling work (P90 in the abstract handbook) related to this study, and found the axon terminals to have the lowest thresholds. While their work is not yet published as full paper, it is worth adding a statement to the discussion of the study limitations here, e.g. that axon terminals of incoming axonal projections were not modelled, but might be further neural elements that are well excitable by TMS.
2. I am still puzzled by the way in which the effect of the TMS field on the neural compartments was modelled (page 5 of the revised manuscript). This seems to depend on the assumption of rotationally symmetric compartments of negligible diameter (as the change of E is only evaluated along the compartment, and the transverse component of E is neglected). In contrast, the soma has a more complex geometry with several branching points at which the axons and the dendritic tree start. As the validity of the main conclusions (i.e. that the axon initial segment close to the soma is most easily stimulated by TMS) depends on an accurate modelling of the TMS effects on this region of the neuron, I would ask the authors to elaborate further on this question. A visualization of the compartmentalization might help to clarify this issue.

I have read this submission. I believe that I have an appropriate level of expertise to confirm that it is of an acceptable scientific standard.

# Response

**Published:** 2017-04-28

**Authors:** Hyeon Seo

**Affiliations:** Gwangju Institute of Science and Technology, Korea, South

1. Thank you for your careful suggestion. We added the statement to the discussion of the study limitations, as follows:

*An important limitation of our study is that we have not included realistic axon morphologies. A thorough modeling of the complex morphologies of local axons could reveal additional sites of activation. In particular, axon terminals whose orientations are aligned with the electric field may be particularly easy to stimulate.*

 2. The compartments are indeed all modeled as cylinders, but with finite diameters. Importantly, that the site of action potential initiation is the axon initial segment does not imply that the electric field necessarily has a strong direct effect on this compartment. There are two effects contributing to action potential initiation at the axon initial segment. First, in response to TMS displaced charges from across the entire dendritic tree can accumulate at the axon initial segment due to the neuronal morphology. Second, due its membrane properties, the axon initial segment is easily excitable. We have clarified these aspects in the discussion, as follows:

 The question of the precise initiation site of action potentials is a central issue for understanding the physiological effects of TMS. According to our study, the dominant initiation site leading to action potentials is the axon initial segment in both L5 and L3 PNs. In previous modeling studies, the action potentials were initiated at the axons crossing the boundary between gray matter and white matter, where the conductivity changes abruptly ^49^ and at the bending parts of the axon due to charge accumulation ^29^ . However Goodwin and Butson (2015) combined a realistic head model with detailed PN models and observed that most action potentials were initiated at or close to the axon hillock just adjacent to the axon initial segment ^28^ . Pashut *et al.* (2011) have also argued for action potential initiation at the axon initial segment ^24^ . Furthermore, this is consistent with previous studies arguing that action potentials giving rise to the D-wave might be initiated close to the soma and/or axon initial segment ^65^^, ^^66^ . *The presence of a highly increased density of fast voltage-gated Na ^+^ and K ^+^ channels leads to high intrinsic excitability of the axon initial segment ^67^. Additionally, the displaced charges from across the entire dendritic tree can accumulate at the axon initial segment due to the neuronal morphology. This means that the initiation of action potentials in the initial segments is not necessarily a direct effect of TMS at that particular site, but rather the consequence of membrane properties and the morphology of PNs.* In our study, L5 PN action potentials were only rarely initiated at the axon where it crosses the boundary between gray matter and white matter, where tissue conductivity changes abruptly ^49^ . It will be important to verify these results with more realistic neuron (in particular: axon) models.

# Peer review: Referee response for version 2

**DOI:** 10.5256/f1000research.10355.r20337

**Published:** 2017-03-24

**Authors:** Socrates Dokos

**Affiliations:** 1 Graduate School of Biomedical Engineering, Faculty of Engineering, University of New South Wales, Sydney, NSW, Australia

The authors have adequately addressed my concerns in their revised manuscript.

I have read this submission. I believe that I have an appropriate level of expertise to confirm that it is of an acceptable scientific standard.

# Peer review: Referee response for version 1

**DOI:** 10.5256/f1000research.9989.r18318

**Published:** 2016-12-07

**Authors:** Socrates Dokos

**Affiliations:** 1 Graduate School of Biomedical Engineering, Faculty of Engineering, University of New South Wales, Sydney, NSW, Australia

1. Please provide a more detailed description in the Methods of how you obtained the primary and secondary electric fields. For instance, you state that the former is directly determined from the coil and head geometries, whilst the latter is calculated using FEM. Why this distinction? Cannot FEM with appropriate boundary conditions be used for a single/combined electric field calculation?
2. Please clarify more clearly the nature of the magnetic dipoles you used to simulate the TMS coil i.e. how many and how were they distributed on the scalp? These dipoles are shown in the panel of Figure 1b as yellow arrows, but I find the figure very unclear, with minimal description in the text.
3. Can you speculate on how the assumption of isotropic conductivity in WM would affect your results? Current is more likely to flow along WM fiber tracts than across these, potentially altering your electric fields and activation results.
4. In the abstract, replace "corctial" with "cortical", and your field calculation methods, replace "in anterior to posterior" with "in the anterior to posterior".

I have read this submission. I believe that I have an appropriate level of expertise to confirm that it is of an acceptable scientific standard, however I have significant reservations, as outlined above.

# Response

**Published:** 2017-01-31

**Authors:** Hyeon Seo

**Affiliations:** Gwangju Institute of Science and Technology, Korea, South

1. The contribution of the proposed approach is combining TMS-induced electric field with the pyramidal neurons. All the process to calculate stimulus-induced electric field using the head model was based on the SimNibs software pipeline, and thus we just briefly introduced the procedure related to field calculations. According to the SimNibs pipeline, they used magnetic dipoles to model the TMS coils as it allows to easily determine the magnetic vector potential of a coil (A-field; the primary electric fields). Furthermore, magnetic vector potential is unaffected by the conductivity in the head model and thus it is straight-forward to calculate. The secondary electric field arises from the charge accumulations at conductivity discontinuities and thus was numerically determined using FEM. For clarity, we now wrote that the field calculation followed the SimNibs pipeline and rephrased corresponding sentences as follows:
  “The TMS-induced electric field was calculated based on the SimNibs v1.1 pipeline. Briefly, the electric field, E→=−∂A→∂t−∇ϕ=−Ep→−Es,→ consisted of primary (Ep→) and secondary (Es→) electric fields. The primary electric field was directly determined by the coil geometry and the secondary electric field caused by charge accumulations at tissue interfaces. Using magnetic dipoles to model the TMS coil, the primary electric field was calculated directly without the volume conductor model and then used as input for the secondary electric field calculation via a finite element method using the GetFEM++ library and MATLAB ^33^^, ^^39^ .”
2. As already responded to the previous comment, we simulated the field following SimNibs software and thus the magnetic dipoles representing the TMS coil was provided in SimNibs. In this paper, the magstim 70 mm figure-8 coil was modelled as two circular disks (radius r = 5 cm) which are divided into 10 rings each (Thielscher and Kammer, 2004.) 
3. In previous study, Opitz *et al.* (2011) investigated the effects of anisotropic conductivity in WM in TMS and revealed the anisotropic conductivity create hot spots in the WM. In addition, Seo *et al.* (2015) investigated the effects of anisotropy on PNs induced by electrical stimulation. Because of this, one could speculate that the anisotropic conductivity may increase activation of L5 PNs. We rephrased these points in the discussion section as follows:
  “There are several limitations in our modeling study. A first limitation is that we have assumed isotropic conductivity, as is common in computational studies. Opitz *et al. *(2011) revealed that anisotropy might create hot spots in the WM with increased field strength that might affect neural excitation ^12^, and Seo *et al. *(2015) reported that anisotropy affected L5 PNs significantly while it had only minor impact on L3 PNs ^45^. Thus anisotropic conductivity might have significant effects on L5 PN axons running through the WM.”
4. Corrected.

# Peer review: Referee response for version 1

**DOI:** 10.5256/f1000research.9989.r15646

**Published:** 2016-09-01

**Authors:** Axel Thielscher

**Affiliations:** 1 Danish Research Centre for Magnetic Resonance, Copenhagen University Hospital Hvidovre, Hvidovre, Denmark

Seo and coworkers combine realistic calculations of the electric field that is induced by TMS with multi-compartmental neural models in order to reveal how and at which sites TMS generates neural activity. Only a few prior studies have targeted this topic in detail, and the presented study contributes relevant new insights into the putative stimulation mechanisms of TMS. Clearly, on the long run, multi-scale modeling approaches as presented by the authors should be superior to approaches based only on field calculations in explaining the biophysics of TMS. As such, I am very supportive of the study and the topic in general. In the following, I suggest changes to improve the clarity of the presentation of the results and to better align them with the known electrophysiological findings for magnetic stimulation of the motor cortex. In addition, I suggest extending the section in the discussion that deals with the study limitations. Given the novelty of the overall approach, it is relevant to educate the readers on the uncertainties involved in the modeling process, and to point toward putative improvements that could be taken up in future studies.

 Comments on the methods and results section (sorted by order of occurrence, not relevance):

- Please specify the origin of the MR data and – if relevant – the underlying ethics approval (page 2, volume conductor model)
- The A field is not dependent on the head model, only coil geometry (page 2, field calculations)
- How much did the re-scaling of the neural models affect the results (page 3, multi-compartmental neuronal models)? This should be tested for a few selected cases. The question how well neuronal models for other species and brain areas can be transferred to human sensorimotor cortex is highly relevant. In that respect, it would be good to know how sensitive the results are towards changes of the local dimensions of the neurons.
- Why were the axons of the L5 PNs modelled to curve towards the corpus callosum rather than the internal capsule (page 3)? How much could this have influenced the results?
- Please give more details on the method used for placing the neurons and axons (page 3). Was it fully automated?
- Adding an external current source to model the TMS field (page 3): How well is this justified for non-cable like structures such as the soma?
- Angular frequency of 30 kHz (page 3): This is too high, 3 kHz would be more realistic. Maybe just a typo?
- Figure 1C: It would be helpful to indicate the region in which the neurons were placed in this plot.
- Figure 3: It is surprising to see that only slight changes in the field strength in dependence on coil orientation were observed. This is in contrast to prior results. The peak field strength might stay relatively constant, but the spatial pattern of the gyral crowns seeing high field strengths should clearly change. Maybe this is only a scaling issue in the plot, or related to the question on where the field was read out (on the CSF-GM surface or within GM?). Please clarify, and compare to prior studies (in particular those using the same FEM method – i.e. simnibs)
- Figure 3 and definition of coil orientations in the text: It would be easier to define the orientations according to the current direction induced in the brain. It is somewhat counterintuitive that that an arrow pointing anteriorly (i.e., in the base orientation) represents a current flowing in the opposite direction, as stated on page 2. Physiological experiments show that M1 has the lowest threshold for monophasic pulses when currents are flowing from posterior to anterior. The results in Figure 4 indicate that this should be the case for the base orientation. This, however, is in contrast to the statement in the methods that current direction was anterior-posterior in that case. Please clarify. A schematic plot of all coil (or better current) directions would be helpful to prevent confusion. In a related manner, please also add information on the meaning of blue and red colors for the lower half of Figure 3 (normal component). Do red colors indicate inflowing or outflowing currents?
- Figure 4: It would be good to be able to see the fields and excitabilities on the medial part of the hand knob. For example, for 270° (L5 PNs), the hand knob seems mostly non-excited. However, this impression might simply result from not being able to see the medial part.
- Figures 4 and 5, and related results: I strongly suggest to differentiate between M1 and S1, and present separate results for both, with a focus on M1. From Figure 5, it seems that coil orientation 135° activates the largest number of neurons. However, Figure 4 indicates that those neurons are mostly in S1. For this reason, it would make more sense to present separate subfigures for M1 and S1 in Figure 5.
- Figure 6, and related results: It seems that axon bends of L5 PNs were never the site where stimulation occurred most easily. This is in contrast to prior hypotheses, and also (simplified) modeling studies. Can you indicate how much higher the excitation thresholds of the bends are? What is the reason that the bends were not excited? By which angle did the axons bend, and which bend radius was modelled?
- Figure 8: It would be good to replace the results using the coil orientation which stimulates the hand knob most strongly, rather than S1.

 

 Comments on the Discussion section:

 This is one of only few studies that combine field calculations with morphologically realistic models of neurons. It shows very encouraging results. Given its pioneering character, however, I would appreciate if the uncertainties involved in the modeling process could be more systematically discussed.I would like to emphasize that this suggestion is not meant as a critique of quality of the study. The match between modeling and experimental results is promising, but should not be misunderstood as a proof that the model is “correct”.

 In addition to limitations in modeling the neurons, uncertainties regarding the tissue conductivities (resulting in uncertainties regarding the field distribution) should be mentioned. The neuron models were taken from cat visual cortex. Even in humans, the histology of visual and motor cortex differs substantially. While the visual cortex has a thick layer 4, the motor cortex is dominated by layer 3 and 5 (including the large “Betz” cells). The study results demonstrate a strong impact of neural features on the results, raising the question on how well a transfer between species and areas is possible. Here, the size of the neurons was rescaled to fit to the target cortical area. I am curious on how much is known about other differences, such as differences in axon and soma diameters, or channel densities, that might systematically impact the results, but where not taken into account? In a related manner, the study of Salvador *et al.* (2011) hints towards putative additional structures that might be affected, such as short- and intermediate connections in WM (their a1 and a2 association fibres), and terminals of incoming fibre projections. It would be worth mentioning them as well.

 I am curious to understand which features of the L3 PNs resulted in their better excitability to TMS? One could intuitively assume that the larger and far-projecting L5 PNs might be the better targets. Discussing this might also help to better reveal the important neural features that dominate the cortical excitability to TMS.

I have read this submission. I believe that I have an appropriate level of expertise to confirm that it is of an acceptable scientific standard, however I have significant reservations, as outlined above.

## References

[1] SalvadorRSilvaSBasserPJMirandaPC: Determining which mechanisms lead to activation in the motor cortex: a modeling study of transcranial magnetic stimulation using realistic stimulus waveforms and sulcal geometry. *Clin Neurophysiol*.2011;122(4) : 10.1016/j.clinph.2010.09.022 748-58 10.1016/j.clinph.2010.09.022 21035390PMC3053122

# Response

**Published:** 2017-01-31

**Authors:** Hyeon Seo

**Affiliations:** Gwangju Institute of Science and Technology, Korea, South

Thank you very much for your valuable comments, which have helped us greatly to improve the manuscript. We reply to your comments point by point below.

  

- *Please specify the origin of the MR data and – if relevant – the underlying ethics approval (page 2, volume conductor model)*

➔ We have used the SimNIBS pipeline to construct the head model and to calculate electric field distributions using the example dataset provided by SimNIBS. We rephrased the corresponding sentences as follows:

 “(At the beginning of Methods section) The volume conductor head model was used to simulate the stimulus-induced electric fields; it was based on the SimNIBS v1.1 software pipeline ^14^^, ^^33^.”

 “(Volume conductor model in Method section) To calculate the precise electric field, a volume conductor head model for TMS that reflected T1-weighted and T2-weighted magnetic resonance (MR) images was constructed using example dataset provided by SimNIBS v1.1 under the ethical approval ^14^”

  

- *The A field is not dependent on the head model, only coil geometry (page 2, field calculations)*

➔ We rephrased the sentence as follows:

 “The primary electric field was directly determined by the coil geometry and the secondary electric field caused by charge accumulations at tissue interfaces.”

  

- *How much did the re-scaling of the neural models affect the results (page 3, multi-compartmental neuronal models)? This should be tested for a few selected cases. The question how well neuronal models for other species and brain areas can be transferred to human sensorimotor cortex is highly relevant. In that respect, it would be good to know how sensitive the results are towards changes of the local dimensions of the neurons.*

➔ We re-scale the dendritic trees while keeping axon and soma identical and observe the spatial distributions of thresholds (Figure 8(b)). We found only slight changes in threshold maps and the percentage of excited PNs by reducing the scale of dendritic trees, while the impact of rotation of dendritic trees was relatively bigger. However, in the presence of morphological changes of dendritic trees, the walls of the precentral gyrus were consistently targeted and the spatial extent of activated regions did not change much. We rephrased the corresponding sentences as follows:

 “(Results) In addition, the impact of scaling of PNs was investigated by reducing the dimension of the dendrite trees by 10%, 20% or 30%, as shown in Figure 8(b). For this, we simply scaled the length of all dendritic compartments while keeping their diameters identical ^46^. We observed consistently activated sites in the threshold maps and only slight changes of the percentage of excited L5 PNs of up to 0.3%. Thus, we found that the rotations of the dendritic trees had a bigger impact on PN excitability than that of scaling the dendritic trees. Overall, morphological changes in dendritic trees did not alter the spatial extent of activated regions much.”

  

- *Why were the axons of the L5 PNs modelled to curve towards the corpus callosum rather than the internal capsule (page 3)? How much could this have influenced the results?*

➔ The corpus callosum was a typo. We tilted the triangular elements comprising the gray matter defined in the ROI toward the internal capsule (Supplementary Figure S1). We rephrased these points as follows:

 “(Multi-compartmental neuronal models in Method section) The axons of L5 PNs were defined to curve beyond the boundary between GM and WM in the direction of the internal capsule (Supplementary Figure S1).”

  

- *Please give more details on the method used for placing the neurons and axons (page 3). Was it fully automated?*

➔ It is not fully automated. When the different neuronal models or different head models are applied, we need to change the parameters to adapt it. We rephrased corresponding paragraphs to include more details as follows:

 “L5/L3 PNs were combined virtually with the head model and modified to accommodate the irregular geometry of the cortex ^28^^, ^^29^^, ^^44^^– ^^47^ , as shown in Figure 2. To reduce superfluous computations, we preselected a region of interest (ROI) of 50 × 50 × 50 mm 3 around the hand knob and then placed L5/L3 PNs in each triangular element comprising the gray matter surface. The multi-compartmental models of PNs consisted of a series of compartments connected by resistors. Each compartment was further discretized into segments of equal length to allow for accurate numerical simulation. The center points of each segment were extracted and used to calculate the necessary changes to neuron geometry, as described below. The dendritic trees were lengthened or shortened by re-scaling the lengths of the compartments according to the local dimensions of the cortex such that dendrites reached layer 1 and the orientation was perpendicular to the cortical surface ^46^^, ^^48^ . Since the morphology of the dendritic trees was not symmetric and it might influence the neuronal activation, L5/L3 PNs had randomly rotated dendritic trees at different locations. The axons of L5 PNs were defined to curve beyond the boundary between GM and WM in the direction of the internal capsule (Supplementary Figure S1). Further adjustments of L5 PNs geometry were as follows (illustrated in Supplementary Figure S2): each dendritic tree was oriented such that its principal axis would align with the normal vector of its associated triangular surface element. The bending part of the axon was calculated according to the normal vector of the surface element. The arc length of the axon bend was set to 0.6 mm when the z-component of the normal vector was positive and otherwise the arc length was 0.3 mm (compare Supplementary Figure S2). Note that when we varied the angle and arc length of the axon bend, it usually did not alter the activation threshold. The axons of L3 PNs were defined to terminate in layer 5/6 within the GM. Altogether, a total of 10,888 L5 PNs and 10,888 L3 PNs was constructed. This process was implemented in MATLAB (MathWorks, Natick, MA, USA).”

  

- *Adding an external current source to model the TMS field (page 3): How well is this justified for non-cable like structures such as the soma?*

➔ All the compartments comprising PNs including the soma were considered by a spatially discretized version of the cable equation, and there are several papers (24. Pashut *et al.*, 2011; 28. Goodwin *et al.*, 2015; 29. Salvador *et al.*, 2011) using the same approach. We are not aware of any problems with adding an external current source in this way.

  

- *Angular frequency of 30 kHz (page 3): This is too high, 3 kHz would be more realistic. Maybe just a typo?*

➔ We corrected it to 30 rad/ms.

  

- *Figure 1C: It would be helpful to indicate the region in which the neurons were placed in this plot.*

➔ We added it.

  

- *Figure 3: It is surprising to see that only slight changes in the field strength in dependence on coil orientation were observed. This is in contrast to prior results. The peak field strength might stay relatively constant, but the spatial pattern of the gyral crowns seeing high field strengths should clearly change. Maybe this is only a scaling issue in the plot, or related to the question on where the field was read out (on the CSF-GM surface or within GM?). Please clarify, and compare to prior studies (in particular those using the same FEM method – i.e. simnibs)*

➔ We changed the color bar scaling, and now the figure reflects coil-orientation changes in the field distribution more clearly.

  

- *Figure 3 and definition of coil orientations in the text: It would be easier to define the orientations according to the current direction induced in the brain. It is somewhat counterintuitive that that an arrow pointing anteriorly (i.e., in the base orientation) represents a current flowing in the opposite direction, as stated on page 2. Physiological experiments show that M1 has the lowest threshold for monophasic pulses when currents are flowing from posterior to anterior. The results in Figure 4 indicate that this should be the case for the base orientation. This, however, is in contrast to the statement in the methods that current direction was anterior-posterior in that case. Please clarify. A schematic plot of all coil (or better current) directions would be helpful to prevent confusion. In a related manner, please also add information on the meaning of blue and red colors for the lower half of Figure 3 (normal component). Do red colors indicate inflowing or outflowing currents?*

➔ Thanks for the suggestion. In the bottom row in Figure 3, red color indicated current directed inwards and blue color means current directed outwards. Thus, in the base orientation, the currents were flowing from posterior to anterior. We corrected the corresponding sentence as follows:

 “(Field calculations) The base coil orientation was defined relative to the direction of the central sulcus that the electric field induced was in the posterior to anterior direction (the yellow arrow in Figure 1(c)).”

  “(Legend in Figure 3) For the orthogonal component of electric fields (bottom row), red color indicates current flowing in the direction from superficial to lower cortical layers and blue color represents currents flowing in the opposite direction.”

  

- *Figure 4: It would be good to be able to see the fields and excitabilities on the medial part of the hand knob. For example, for 270° (L5 PNs), the hand knob seems mostly non-excited. However, this impression might simply result from not being able to see the medial part.*

➔ We made Supplementary Figure S3 (an animated gif) to show the medial part of the hand knob.

  

- *Figures 4 and 5, and related results: I strongly suggest to differentiate between M1 and S1, and present separate results for both, with a focus on M1. From Figure 5, it seems that coil orientation 135° activates the largest number of neurons. However, Figure 4 indicates that those neurons are mostly in S1. For this reason, it would make more sense to present separate subfigures for M1 and S1 in Figure 5.*

➔ We appreciate the reviewer’s suggestion. We separated results for precentral and postcentral gyrus, as shown in Figure 5 (for Figure 4, we already showed spatial distributions of thresholds separately for the precentral and postcentral gyrus). We rephrased corresponding sentences in Results and Discussion sections, as follows:

 “(Results section) The percentage of excited neurons for a stimulation intensity at the motor threshold is shown in Figure 5. We separately analyzed neurons falling in the precentral and postcentral gyrus. When we focused on PN activations in the precentral gyrus, the highest percentage of excited neurons was observed at +90 degrees rather than the base orientation. For the base orientation PNs in the sulcal wall along the central sulcus were activated. At +90 degrees PNs were activated mostly in the opposite sulcal wall (Supplementary Figure S3). For the postcentral gyrus, the maximum percentage of activated PNs was observed when the coil was oriented at +135 degrees, which is in agreement with the threshold maps in Figure 4.”

  

- *Figure 6, and related results: It seems that axon bends of L5 PNs were never the site where stimulation occurred most easily. This is in contrast to prior hypotheses, and also (simplified) modeling studies. Can you indicate how much higher the excitation thresholds of the bends are? What is the reason that the bends were not excited? By which angle did the axons bend, and which bend radius was modelled?*

➔ According to [28] Goodwin *et al.* (2015) who combined a realistic head model with pyramidal neurons, most neurons initiated action potentials at close to or within the axon hillock just adjacent to the axon initial segment, and [24] Pashut *et al.* (2011) who investigated the neuronal responses induced by magnetic stimulation also argued that action potential initiation is in the axon initial segment. Thus, these recent modeling studies reported the axon initial segment as action potential initiation site. The different observation compared to the results using simplified models might be induced because of different morphology and electrical properties of incorporated PNs. As we mentioned in the Discussion section, further study of the impact of neuronal morphology and electrical properties will be helpful. We rephrased corresponding sentences as follows:

 “(Discussion section) The question of the precise initiation site of action potentials is a central issue for understanding the physiological effects of TMS. According to our study, the dominant initiation site leading to action potentials is the axon initial segment in both L5 and L3 PNs. In previous modeling studies, the action potentials were initiated at the axons crossing the boundary between gray matter and white matter, where the conductivity changes abruptly ^4^ ^9^ and at the bending parts of the axon due to charge accumulation ^29^. However, Goodwin *et al.* (2015) combined a realistic head model with detailed PN models and observed that most action potentials were initiated at or close to the axon hillock just adjacent to the axon initial segment. Pashut *et al.* (2011) have also argued for action potential initiation at the axon initial segment. Furthermore, this is consistent with previous studies arguing that action potentials giving rise to the D-wave might be initiated close to the soma and/or axon initial segment ^65^^, ^^66^ . In our study, L5 PN action potentials were only rarely initiated at the axon where it crosses the boundary between gray matter and white matter, where tissue conductivity changes abruptly ^49^ . It will be important to verify these results with more realistic neuron (in particular: axon) models.”

 ➔ In addition, when we control the arc length from 0.6 mm over 0.3 mm to 0 mm we found that the threshold did not alter as long as the bend was smooth, while a bend with 0 mm arc length produced an increased threshold. In this work, 0.6 mm arc length was determined to reproduce the results from Wongwarnpigoon *et al.*, 2012 that investigated the impact of epidural cortical stimulation using same pyramidal neurons. We added text how the axon bending parts were made as follows:

 “(Method – Multi-compartment neuronal models) The bending part of the axon was calculated according to the normal vector of the surface element. The arc length of the axon bend was set to 0.6 mm when the z-component of the normal vector was positive and otherwise the arc length was 0.3 mm (compare Supplementary Figure S2). Note that when we varied the angle and arc length of the axon bend, it usually did not alter the activation threshold.”

  

- *Figure 8: It would be good to replace the results using the coil orientation which stimulates the hand knob most strongly, rather than S1.*

➔ We appreciate the reviewer’s suggestion. We changed Figure 8 to the case for the base coil orientation.

*Comments on the Discussion section:*

**

*This is one of only few studies that combine field calculations with morphologically realistic models of neurons. It shows very encouraging results. Given its pioneering character, however, I would appreciate if the uncertainties involved in the modeling process could be more systematically discussed.I would like to emphasize that this suggestion is not meant as a critique of quality of the study. The match between modeling and experimental results is promising, but should not be misunderstood as a proof that the model is “correct”.*

 ➔ A critical limitation involved in the proposed modeling process is that straightly stretched axons of L5 PNs inside the WM are not realistic. Thus, as we mentioned in discussion section, further developments in tractography using DTI may improve detailed neuronal morphology and thus we expect that more realistic PNs can be constructed in the future. The other uncertainties of the neuronal models were related to electrical and morphological variation among and within PNs. These issues were addressed below according to the next comments.

 In addition to limitations in modeling the neurons, uncertainties regarding the tissue conductivities (resulting in uncertainties regarding the field distribution) should be mentioned. The neuron models were taken from cat visual cortex. Even in humans, the histology of visual and motor cortex differs substantially. While the visual cortex has a thick layer 4, the motor cortex is dominated by layer 3 and 5 (including the large “Betz” cells). The study results demonstrate a strong impact of neural features on the results, raising the question on how well a transfer between species and areas is possible. Here, the size of the neurons was rescaled to fit to the target cortical area. I am curious on how much is known about other differences, such as differences in axon and soma diameters, or channel densities, that might systematically impact the results, but where not taken into account? In a related manner, the study of Salvador *et al.* (2011) hints towards putative additional structures that might be affected, such as short- and intermediate connections in WM (their a1 and a2 association fibres), and terminals of incoming fibre projections. It would be worth mentioning them as well.

 ➔ We agree that neural features have a strong impact on the results. In this work, we only varied the morphology of PNs (L3 and L5 PNs). However, further variation regarding to morphological and electrical properties should be considered. Thus, we rephrased the corresponding sentences as follows:

 “There are several limitations in our modeling study. A first limitation is that we have assumed isotropic conductivity, as is common in computational studies. Opitz *et al.* (2011) revealed that anisotropy might create hot spots in the WM with increased field strength that might affect neural excitation ^12^, and Seo et al (2015) reported that anisotropy affected L5 PNs significantly while it had only minor impact on L3 PNs ^45^. Thus anisotropic conductivity might have significant effects on L5 PN axons running through the WM.

 In this work, the L3 and L5 PNs were taken from cat visual cortex due to the lack of models for most human cortical cell types. Thus, while we lengthened PNs to fit the cortex, the uncertainties regarding morphology of neurons was not fully studied. Wu *et al.* (2016) incorporated a multitude of PNs with various stimuli ^25^ and Salvador *et al.* (2010) constructed various types of neural structures including pyramidal neurons, interneurons, and association fibers ^29^; they found that the excitability can be shaped by field orientation, pulse wave form, and diameter of neurons. In addition, changes in the electrical properties, such as membrane properties and ion channels had the largest influence on neuronal excitability ^25^. However, despite the uncertainty with regard to properties of PNs, we produced results matching both experimental studies and other computational studies ^23^^, ^^25^ that incorporated the same models of PNs.”

*I am curious to understand which features of the L3 PNs resulted in their better excitability to TMS? One could intuitively assume that the larger and far-projecting L5 PNs might be the better targets. Discussing this might also help to better reveal the important neural features that dominate the cortical excitability to TMS.*

 ➔ Mainen and Sejnowski, 1996, [41], tested the influence of dendritic structure by constructing neurons that share a common distribution of ion channels and differ only in their dendritic geometry (Fig 1). In that study, we used Fig.1 (c) morphology for L3 PNs and Fig 1. (d) morphology for L5 PNs. Their intracellular thresholds to evoke action potentials are 0.2 nA for L5 PNs and 0.1 nA for L3 PNs. From this, we can see that L3 PNs had a lower threshold, i.e. better excitability, compared to L5 PNs. In addition, better excitability to invasive cortical stimulation was also observed in previous modeling studies (ref [45] and [56]). It is plausible therefore that the morphology of L3 PNs might contribute to their better excitability to TMS. We rephrased the corresponding sentences as follows:

  “In this work, we explored the excitation thresholds of both L3 and L5 PNs and found as another major result that the percentage of excited neurons for a stimulation intensity at the motor threshold was about two times higher for L3 PNs than for L5 PNs. Furthermore, the activation of the L3 PNs was consistently higher than that of the L5 PNs for the full range of stimulation intensities. Mainen and Sejnowski (1996)  compared the dendritic structure of L3 and L5 PNs with a common distribution of ion channels and found that a smaller intracellular current injection was necessary to activate L3 PNs compared to L5 PNs ^42^. Thus, the morphology of L3 PNs might result in their higher excitability in response to TMS. In addition, the lower stimulation thresholds of the L3 PNs are consistent with lower stimulation intensities required to produce I-waves ^50^^, ^^62^ , and the higher stimulation intensities required to produce D-waves.”
